# Immunodetection of NETs in Paraffin-Embedded Tissue

**DOI:** 10.3389/fimmu.2016.00513

**Published:** 2016-11-22

**Authors:** Volker Brinkmann, Ulrike Abu Abed, Christian Goosmann, Arturo Zychlinsky

**Affiliations:** ^1^Microscopy Core Facility, Max Planck Institute for Infection Biology, Berlin, Germany; ^2^Department of Cellular Microbiology, Max Planck Institute for Infection Biology, Berlin, Germany

**Keywords:** NETs, immunodetection, paraffin-embedded tissue, image analysis, antigen retrieval

## Abstract

The pathogenic potential of neutrophil extracellular traps (NETs) was recently described, and their detection in tissue could serve as a prognostic marker. NETs are delicate and filigree structures; hence good tissue preservation is essential for their detection. Indeed, analysis of paraffin-embedded tissue has proven superior to the study of cryo sections. Though, under favorable conditions, the presence of NETs can be detected in tissue sections stained with histological dyes, definitive identification of NETs needs the colocalization of immunofluorescent signals for both nuclear and granular (or cytoplasmic) NET components. We tested diverse antigen retrieval methods and various combinations of commercially available antibodies and present here staining protocols to detect NETs in human and murine tissue sections.

## Introduction

Neutrophil extracellular traps (NETs) probably evolved to counteract invading pathogens (1). When their production or degradation is not controlled, they have pathogenic potential and are involved in numerous diseases including autoimmunity, thrombosis, lung diseases, infertility, diabetes, cancer, and neurodegeneration, reviewed in (2). NETs are composed of chromatin, granular, and some cytoplasmic constituents (3). In naive neutrophils, granular and nuclear antigens are spatially separated and during NETosis, some granular proteins, like neutrophil elastase (NE) and myeloperoxidase (MPO) gradually migrate to the nucleus (4, 5). Also, histones are citrullinated during NETosis (6, 7). Thus, the localization of granular proteins in the nucleus and the citrullination of histones provide unique feature for neutrophils undergoing NETosis that can be exploited for identification of these cells in tissue sections.

*In vitro*, there are a variety of protocols to detect and quantify NETs (8–12). It is important to note that the widely spread out strands associated with NET images generated *in vitro* are probably an artifact of fixation. Indeed, in life cell imaging studies with isolated neutrophils, NETs appear as a diffuse cloud formed by the strands of NETs floating in the medium (13). It is not clear how NETs appear in tissues, where space is restricted, and the NETs are unlikely to appear as the large areas observed *in vitro*. Classical histological stains, like hematoxylin/eosin, may indicate the presence of NETs, but there are only few examples of NETs detection with histological stains (14, 15). Notably, there is no general protocol to identify NETs in tissues.

While they are well suited for staining with most antibodies, cryo sections from freshly frozen tissue have the disadvantage that due to ice crystal formation neutrophils in the tissue can be damaged, thus NET-like structures can be generated as a preparation artifact. In contrast, fixation with buffered paraformaldehyde solution, ideally by perfusion, preserves the tissue architecture including NETs. Here, we present methods for formalin-fixed and paraffin-embedded sections. These sections are available from pathological studies and can be conserved indefinitely before analysis.

Most antibodies will not readily bind to their epitopes in formalin-fixed tissue. The reason for this is the formation of intra- and intermolecular cross-links by methylene bridges that mask most epitopes (16, 17). For successful immunohistological staining, antigen retrieval is required that normally involves heating of the rehydrated sections in a suitable heat-induced epitope retrieval (HIER) buffer (18, 19). This breaks the methylene bridges that prevent binding of the antibody and renders the epitopes accessible. In a study with histopathologically important antibodies, it was shown that most epitopes detected by clinically relevant antibodies are linear and can be reversibly blocked by binding to neighboring proteins during fixation (20).

We tested a series of antibodies against NET components for their ability to bind to their epitopes in formalin-fixed paraffin-embedded tissue. We selected nine antibodies with good staining properties and tested various antigen retrieval methods to find suitable combinations for double or triple immunofluorescence. In this paper, we present protocols that allow simultaneous staining for nuclear and granular or cytoplasmic NET components in paraffin-embedded tissue sections after antigen retrieval.

For the identification of NETs it is necessary to determine if nuclear antigens are colocalized with granular and/or cytoplasmic components. Hence, micrographs have to be prepared at a primary magnification of at least 20×. The resulting images can be used to quantify the fluorescent signals as an unbiased means for the detection and measurement of NETs in tissue.

## Materials and Equipment

Archived paraffin blocks of mouse lungs and of a human brain fungal abscess were used. Mouse breeding and experiments were approved by the Berlin state authority Landesamt für Gesundheit und Soziales, permit G0200/15. Pathology sample collection was approved by the ethical committee of Charité University Hospital, Berlin, Germany.

All antibodies were obtained from commercial suppliers (Table 1). The following antigen retrieval solutions were used: R-Universal Buffer pH7, 10× (Aptum APO 0530500), Target Retrieval Solution pH9 10mM Tris (TRS) 10× (Dako S236784), and Target Retrieval Solution pH6 10mM Citrate 10× (Dako S236984-3).

**Table 1:** **Overview of the antibodies that allow immunostaining for NET components in paraffin-embedded tissue and the respective most effective antigen retrieval protocol**.

Source	Clone	Specificity	Host dilution		37°C		50°C	50°C		60°C		96°C	
					Citrate pH6	TRS pH9	R-Univ pH7	Citrate pH6	TRS pH9	Citrate pH6	TRS pH9	Citrate pH6	TRS pH9
Abcam ab134211	–	Histone H2B	ck 1:500	H2B	++^1^ N	+/−	++^1^ N	++^1^ N Q	++^1^ N	+++ N	++^1^ N	++ N	+++ N
Antibodies-Online ABIN1735464	–	Histone H3	sh 1:100	H3	+/−	+/− N	++^1^ N (Q)	++^1^ N Q	++^1^ N Q	++^1^ N (Q)	++ N	++ N	++^1^ N
Abcam ab5103	–	Histone H3 citrulline (R2 + R8 + R17)	rb 1:50	H3cit	++^1^ (N)	+/−	++ N Q	++ N Q	++^1^ (N) Q	+/− N	+ (N)	+++ N	+++ N
Millipore 481001	–	Neutrophil elastase	rb 1:50	NE	++ (N)	+/−	++ N	++ N	++ (N)	+ (N)	−	−	−
LS-B4244	–	HN elane^a^	sh 1:200	NEsh	++	++	++	++ N	++ N	++	++	−	−
R&D Systems AF3667	–	Myeloperoxidase	gt 1:200	MPO	++	++	++	++	++	++	++	++	++
Biorbyt orb316605	–	CalgrA S100A8 MRP14	rb 1:200	CalA	+++ N	+++ N	+++ N	+++ N	+++ N	+++ N	+++ N	+++ N	+++ N
Biorbyt orb315186	–	CalgrB S100A9 MRP14	rb 1:200	CalB	+++ N	+++ N	+++ N	+++ N	+++ N	+++ N	+++ N	+++ N	+++ N
BDPharmingen 551459	1A8	Ly6G	rt 1:200	Ly6G	++	+++	+++	+++	+++	+++	+++	+++	+++

*^a^Only for human tissue*.

*R-Universal Buffer pH7 (RUB) 10× Aptum APO 0530500*.

*Target Retrieval Solution pH9 10mM Tris (TRS) 10× Dako S236784*.

*Target Retrieval Solution pH6 10mM Citrate 10× Dako S236984-3*.

*N, NET visualizations*.

*+++, strong staining of expected epitopes*.

*++, satisfying specific positive result*.

*+, moderate specific positive result*.

*+/−, weak or partially positive*.

*−, completely negative result*.

*Q, suitable as NET marker for quantification under mentioned conditions*.

*^1^, decondensed nuclei+++, nuclei +/−, or +*.

*(), limitations*.

Fluorescence images were recorded using a Leica SP8 confocal or a Leica DMR widefield microscope (equipped with bandpass filter blocks and a Jenoptik ProgRes MF USB camera).

Complete tissue sections were digitized using a ZEISS Axioscan Z1 slide scanner.

## Stepwise Procedures

### Immunofluorescence of Tissue Sections

The mouse tissue had been fixed *in situ* by transcardial perfusion with 2% paraformaldehyde solution in TRIS-buffered saline (TBS, pH 7.4). Following this, the lungs were carefully removed and post-fixed in 2% paraformaldehyde for 16–20 h at RT. The tissue was then dehydrated and paraffin-embedded (60°C) using a Leica TP 1020 tissue processor. Human brain fungal abscess tissue was from archived paraffin blocks; fixation conditions are not known.

Paraffin blocks were cut at 3 μm, sections were mounted and dried on Superfrost Plus slides (Thermo Scientific) avoiding temperatures above 37°C. After dewaxing and rehydration, sections were incubated in one of the HIER buffers at different temperatures [20 min at 96°C in a steam cooker (Braun) or 90 min at lower temperatures in a water bath, details in Table 1].

After antigen retrieval, sections were left in the respective HIER buffer at RT to cool below 30°C, rinsed with deionized water three times, TBS pH7.4 one time, and permeabilized for 5 min with 0.5% Triton X100 in TBS at RT, followed by three rinsing steps with TBS.

Sections were surrounded with PAP-pen and treated with blocking buffer for 30 min to prevent non-specific binding. Primary antibodies (Table 1) were diluted in blocking buffer and incubated on the sections over night at 37°C. At any one time, two or three primary antibodies requiring the same antigen retrieval protocol raised in different hosts were combined. We used secondary antibodies raised in donkey and pre-absorbed against serum proteins from multiple host species (Jackson ImmunoResearch). Dilution and blocking buffer was TBS supplemented with 1% BSA/2% donkey NS/5% cold water fish gelatin/0.05% Tween 20/0.05%Triton X100.

### Hematoxylin/Eosin Histology

Consecutive sections were stained with hematoxylin/eosin using standard protocols.

### Image Analysis

Image sets were analyzed using the Fiji-ImageJ software package (21) and a common spreadsheet application. The FigureJ plugin (22) was used to assemble Figures 2 and 3.

## Results

### Suitable Combinations of Antibodies and Antigen Retrieval Methods

**Figure 1:**
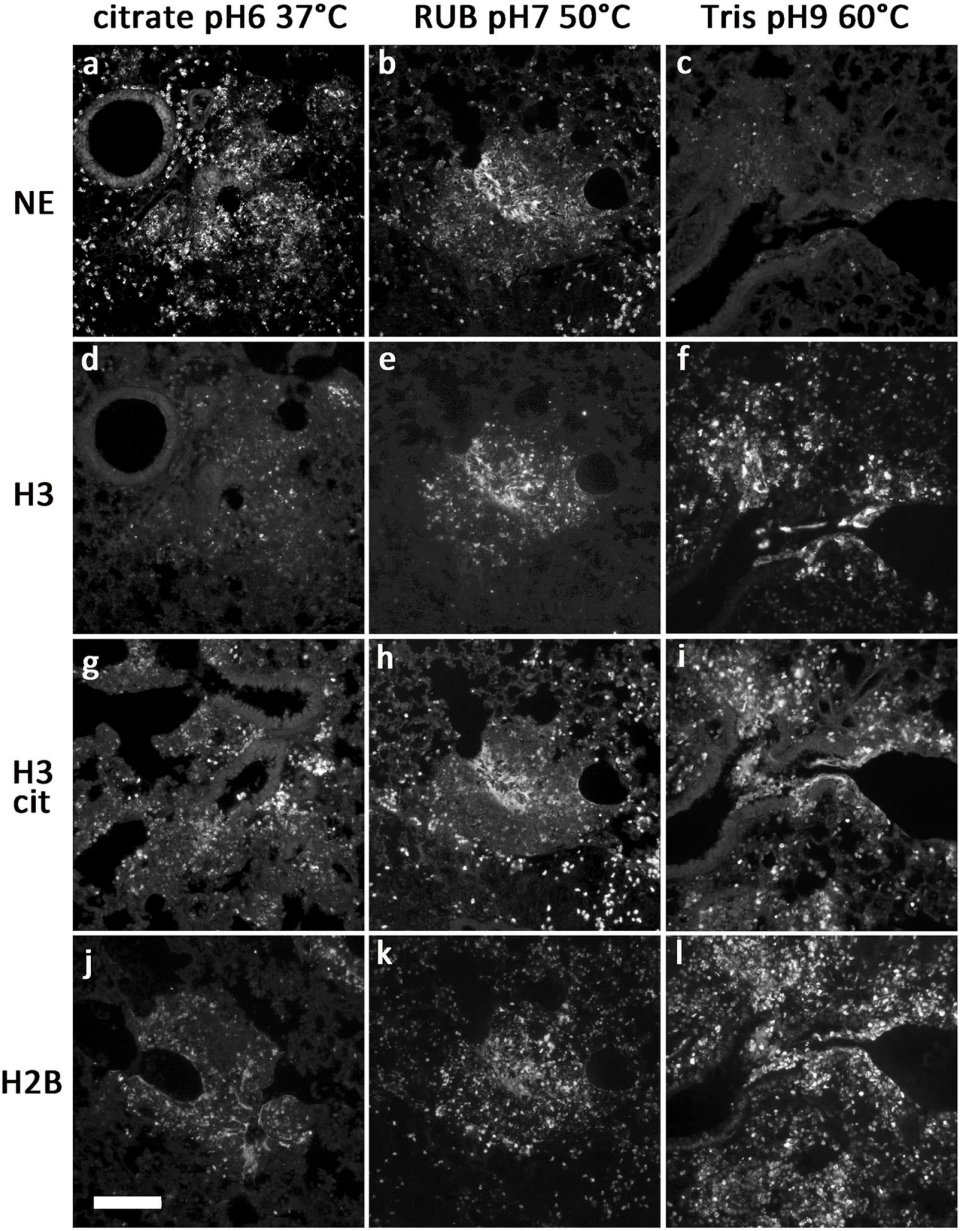
**Representative images of antibody stainings in sections of a paraffin-embedded *Candida albicans*-infected mouse lung**. Different antigen retrieval methods were used (details in Table 1). Antibodies were against NE **(A–C)**, H3 **(D–F)**, citrullinated H3 **(G–I)**, and H2B **(J–L)**. Bar represents 100 μm.

Table 1 summarizes the results of immunofluorescence staining for NET components and neutrophil marker proteins under different antigen retrieval conditions. Figure 1 shows immunofluorescence images resulting from different antigen retrieval protocols. Immunodetection of NE is dependent on the incubation temperature, which may not exceed 60°C (Figure 1C). Staining is stronger at pH6 compared to pH9. The cytoplasmic NET component calprotectin (3), a heterodimer consisting of Calgranulin A and B, is readily detected at all temperatures tested. As expected, the staining patterns for both subunits did not differ.

We tested various antibodies against histones. Only the ones that gave reliable immunostaining with NETs are specified in Table 1. Interestingly, both antibodies against H3 and H2B produced different staining patterns depending on the antigen retrieval temperature. Incubation of the sections at temperatures above 55°C resulted in a strong staining of NETs and nuclei (Figures 1F,I). In contrast, at temperatures between 37 and 50°C, both antibodies reacted predominantly with relaxed chromatin in netting neutrophils and NETs, while normal neutrophils and other cells show a weak nuclear staining (Figures 1D,E,J,K; also Figure 3A). A similar staining pattern has been described for an antibody against a subnucleosomal complex in NETs derived *in vitro* from isolated neutrophils (13). Taken together, this difference in staining is probably due to the compaction of chromatin and the state of the antigen detected. Importantly, antibodies against citrullinated H3 (H3cit) reacted at all temperatures tested, and the staining pattern was nearly exclusively in areas with netting neutrophils and NETs (Figures 1G–I) (6, 7).

**Figure 2:**
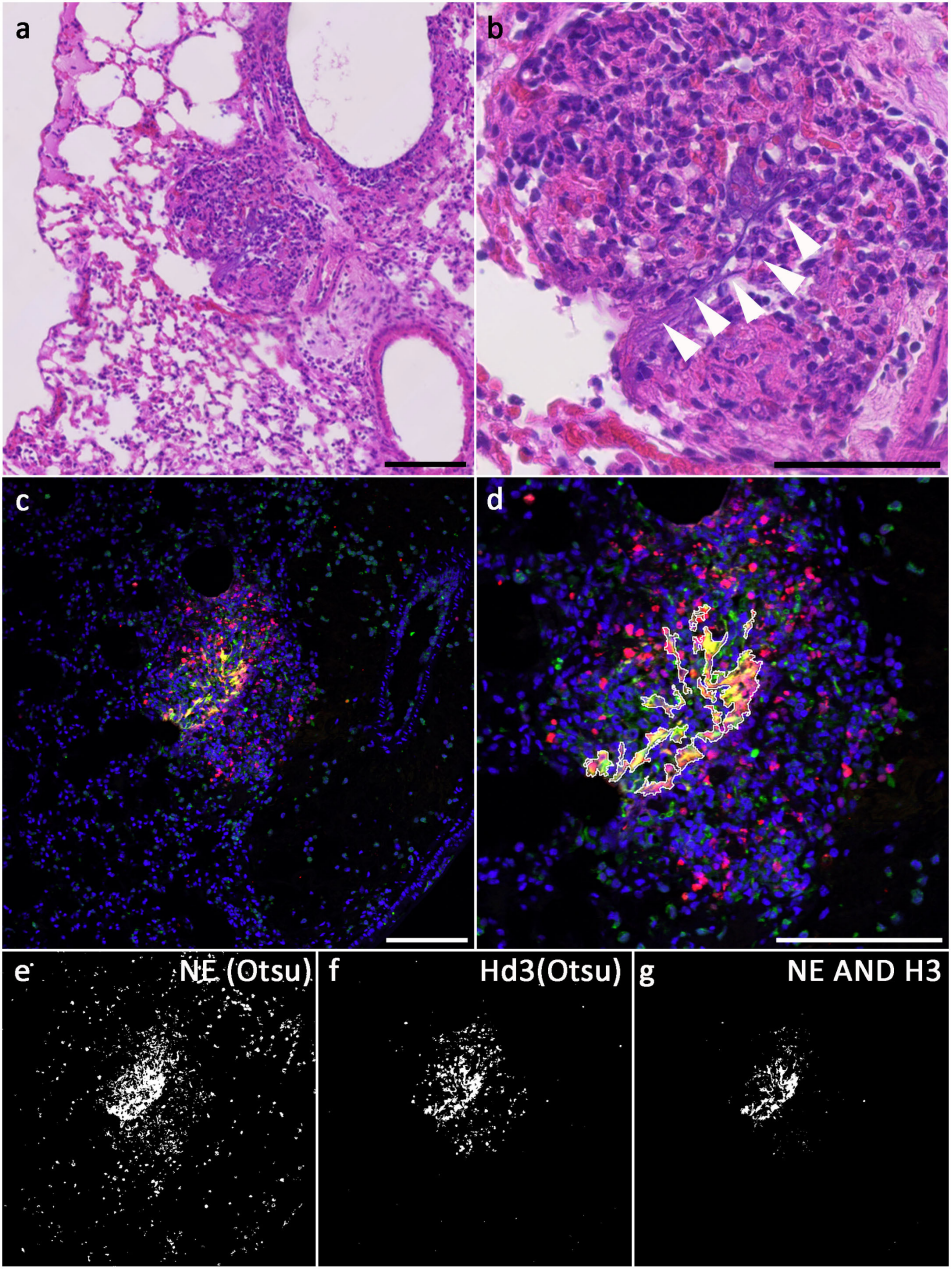
**NETs in a *Candida*-infected mouse lung; (A,B) hematoxylin/eosin staining of a *Candida albicans*-infected mouse lung, consecutive section to the one used in (C–G)**. Areas of extracellular DNA are present (arrowheads), which stain for NET markers **(C)**. **(C)** Merged fluorescence images showing DNA: blue, NE immunolabel: green, H3 immunolabel: red; **(E,F)** binarized images after Otsu thresholding, **(E)** NE above threshold, **(F)** H3 above threshold, **(G)** NETs [intersection of both, NE ∧ H3; outline of this area is superimposed in **(D)**]; **(D)** magnified section of the merged fluorescence image with outline of NETs segmentation (excluding areas <30 pixels). Bars represent 100 μm.

To clearly identify NETs in tissue, colocalization of granular and nuclear components has to be detected. We chose antibodies against NE and either H3 or H2B in combination with detection of citrullinated H3. As a compromise for the different conditions of antigen retrieval, we chose Buffer R-Universal at neutral pH, which allows simultaneous immunodetection of histones and NE. At magnifications of 20× or higher, the resulting images can be used for automatic detection of NET-containing areas in tissue (Figure 2C).

### Segmentation of Areas Positive for Nuclear and Granular NET Components Allow Quantification of NETs in Tissue

Figure 2C shows a confocal image of a neutrophil-rich area of a mouse lung infected with *Candida albicans* and stained for NE (green, Millipore 48101) and H3 (red, ABIN 1735464) as well as for DNA (Hoechst 33342). Antigen retrieval was performed with R-Universal Buffer at 50°C. Using automatic Otsu thresholding, positive areas for both channels were depicted white, while areas below threshold were depicted black (Figures 2E,F). The overlap of both indicating the NET-positive area is shown in Figure 2G and superimposed on the tissue staining (Figure 2D).

Hematoxylin/eosin staining of the same area in a consecutive section is presented in Figures 2A,B. The overview shows infiltration of neutrophils (Figure 2B, center). At higher magnification, extracellular strands of DNA are visible (arrowheads in Figure 2B). Identification of these strands as NETs needs immunofluorescence.

### Staining Pattern of Antibodies against H3 and H2B Is Dependent on the Antigen Retrieval Protocol

**Figure 3:**
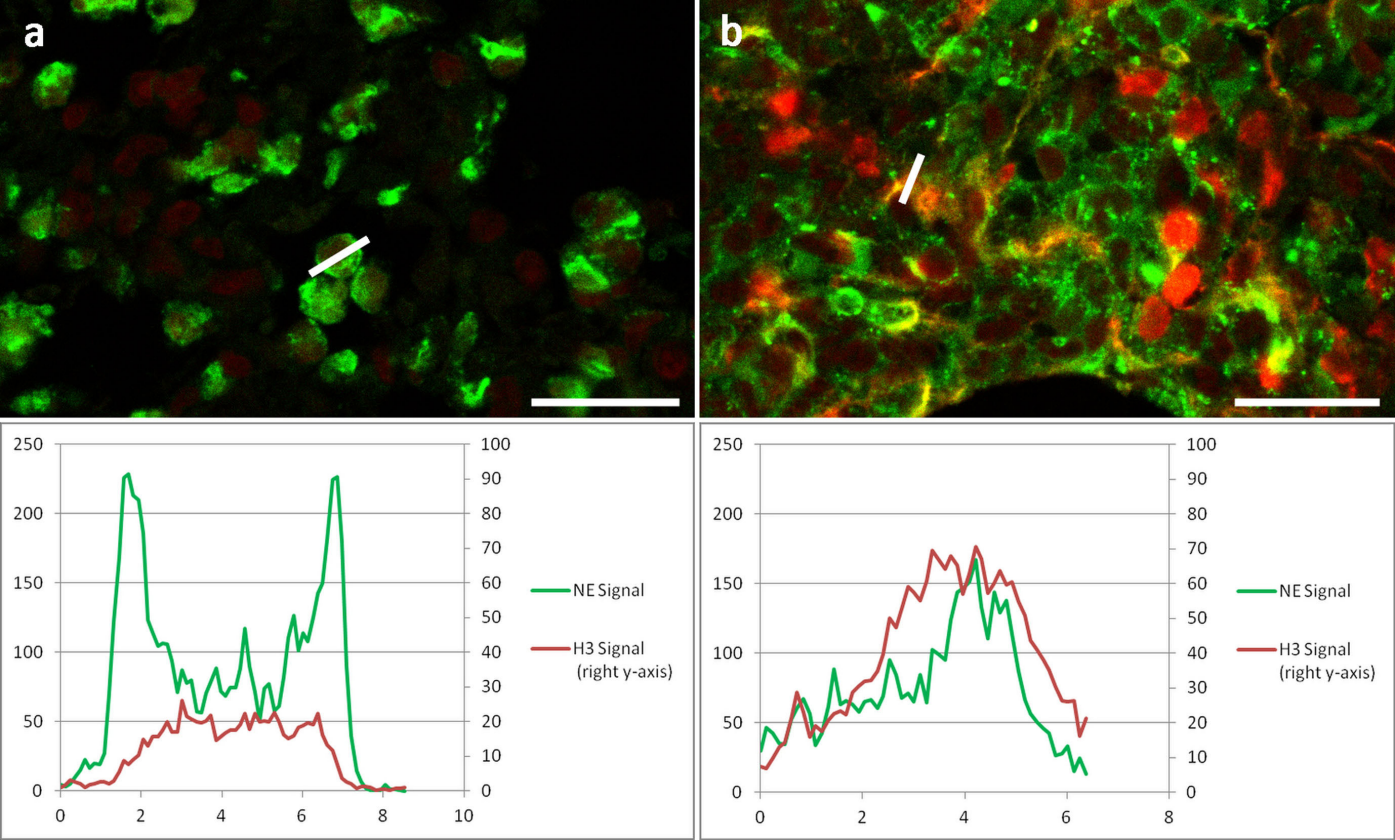
**Two areas of the same section of a *Candida albicans*-infected mouse lung at higher resolution showing NE immunolabel: Green, H3 immunolabel: red**. **(A)** Area with non-NETotic Neutrophils with a line profile of a neutrophil granulocyte. **(B)** Area with NETs with line profile of a NET; both areas were imaged with identical settings. Below images are the respective fluorescence intensity plots of the line profiles for both channels. Bar represents 20 μm.

We further analyzed the staining pattern of anti-histone antibodies. When antigen retrieval was accomplished with incubations above 55°C, a strong staining of all nuclei and NETs was detected (Figures 1F,I). In contrast, only decondensed nuclei of neutrophils as well as NETs stained strongly with these antibodies if the antigen retrieval incubation did not exceed 50°C (Figure 3B). Images recorded under identical conditions of normal and netting neutrophils in the same tissue section revealed clear differences in fluorescence brightness. While H3 fluorescence in normal neutrophils rarely reached intensity values of 60 (red channel in Figure 3A), using identical settings, intensity in areas with NETosis often reached saturation (256, Figure 3B). Representative intensity line profiles are presented below the micrographs including the intensity values for NE (green). As expected, in normal neutrophils, highest NE intensity was found surrounding the nuclear area (in the cytoplasm), as opposed to netting neutrophils showing similar line plots for NE and H3 indicating colocalization of nuclear and granular NET components.

### Immunodetection of NETs in Archived Human Tissue

**Figure 4:**
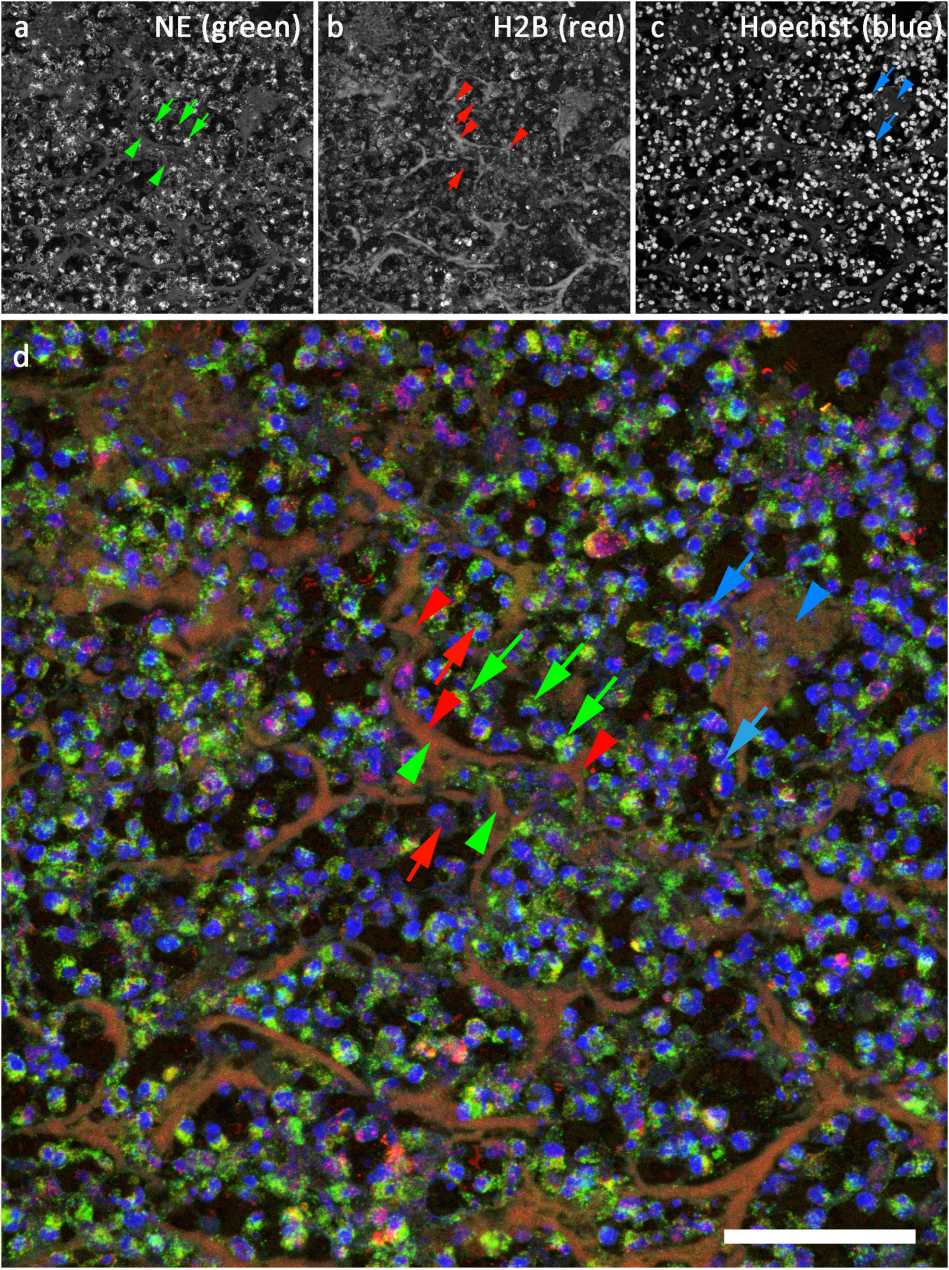
**Confocal image of NETs in an archived sample of human brain fungal abscess**. **(A)** Staining for neutrophil elastase (LS-B4244). Green in **(D)**. **(B)** Staining for histone 2B (Abcam ab134211), red in **(D)**. **(C)** DNA stain (Hoechst 33342), blue in **(D)**. Bar represents 50 μm.

The tissue section (archived human brain fungal abscess) depicted in Figure 4 was stained with antibodies against NE (LS-B4244, Figure 4A, and green in Figure 4D) and against Histone 2B (H2B, Abcam ab 134211, Figure 2B, and red in Figure 4D) after antigen retrieval using R-Universal Buffer at 50°C. DNA was stained with Hoechst 33342 (Figure 4C and blue in Figure 4D). NE-staining shows a very strong granular signal (green arrows in Figures 4A,D) compared to the rather faint diffuse staining in NETs (green arrowheads in Figures 4A,D). Conversely, the signal for H2B is stronger in NETs (red arrowheads in Figures 4B,D) than in compact nuclei (red arrows in Figures 4B,D). This pattern of strong histone staining in NETs but weak staining in compact nuclei is similar to that of H3 described for staining in mouse tissue (Figure 3). DNA staining is strongest at areas with high DNA concentration (compact nuclei, blue arrows in Figures 3A,B), while relaxed chromatin in NETs shows a diffuse staining (blue arrowheads in Figures 3A,B).

## Discussion

Due to their pathogenic potential, identification of NETs in tissue samples both from patients and from laboratory animals is important and could be of diagnostic value. In contrast to staining of NETs derived from isolated neutrophils stimulated *in vitro*, NETs in tissue are not easily identified, and staining for just one component, e.g., DNA, is not sufficient.

In order to allow simultaneous immunodetection of two or three NET components, we tested a series of commercially available antibodies for their property to react with NET markers in paraffin-embedded tissue. Each antibody was tested using various antigen retrieval protocols (Table 1). We use unlabeled primary antibodies from different hosts and detect the bound antibodies with species-specific secondary antibodies, which are cross-absorbed against serum proteins of a number of hosts. This avoids false-positive staining due to unspecific cross-labeling and facilitates dye swaps using different combinations of secondary antibodies. We found that good results were obtained performing antigen retrieval at neutral pH and temperatures between 37 and 50°C, which allow combinations of various antibodies against nuclear, granular, and cytoplasmic NET components (Figure 1).

In resting neutrophils, these are clearly segregated, and immunofluorescence staining reveals no overlap of the signals if magnifications of 20× or higher are used given that the section thickness does not exceed 3–5 μm or confocal microscopy is used. During NETosis, NET components gradually intermingle to a homogenous mixture in late phases of NETosis and in NETs. Accordingly, fluorescence signals for nuclear and granular or cytoplasmic NET components overlap increasingly. These fluorescence signals can be segmented automatically (Figures 2C,D), and the area of signal overlap defines NETs (Figures 2B,E). In hematoxylin/eosin-stained tissue slices, NETs can appear as dark diffuse strands [Figures 2F,G and Ref. (14)], but the positive identification of these smears demands overlapping immunodetection of NET components.

We found that under mild antigen retrieval conditions, antibodies against H3 (Figure 3) and H2B (Figures 4B,D) stain relaxed chromatin as present in NETs and netting neutrophils stronger than the compact chromatin of normal nuclei. This property can be used to scan at low power magnifications for areas that may contain NETs for subsequent detailed analysis using a second NET marker.

Figure 4 depicts a section of an archived sample of human brain fungal abscess stained for NE (Figure 4A, green in Figure 4D), H2B (Figure 4B, red in Figure 4D), and DNA (Figure 4C, blue in Figure 4D). Interestingly, the staining intensity for NE is very high in granules but rather low in NETs. This is probably due to differences in protein concentration. In contrast, staining for H2B is generally lower in condensed nuclei than in NETs. Presumably, the epitope of this antibody is better accessible in relaxed compared to compact chromatin. Notably, this preference for binding to decondensed chromatin depends on the temperature used for antigen retrieval: when HIER buffer is heated above 55°C, antibodies against H2B and H3 react strongly both with NETs and with compact nuclei. Apparently, antigen retrieval at higher temperatures exposes histone epitopes that are normally hidden.

Ly6G is a differentiation antigen, which is expressed in mature neutrophils. In areas of massive infiltration, neutrophils are densely packed leaving nearly no space between the cells. Under these conditions, GPI-anchored Ly6G delineating the cell membrane can come in close contact to extracellular NETs that may result in the interpretation of Ly6G-positive areas as NETs (23). It has been shown that NETs do not contain membrane proteins, and Ly6G was not found as a NET constituent (3). For proper identification of NETs in tissue, immunostaining for generally accepted NET markers as presented in this protocol should be employed.

We have identified a set of antibodies, which can be used to detect NET components in paraffin-embedded tissue both of human and murine origin. Using mild antigen retrieval protocols, many of these antibodies can be combined to yield a satisfactory signal intensity. We hope that these protocols will be useful for a more reliable detection of NETs in tissue.

## Author Contributions

Designed study: VB, UA, CG, and AZ. Tissue immunostainings: UA. Microscopy: UA and VB. Image analysis: CG. Wrote the manuscript: VB, UA, CG, and AZ.

## Conflict of Interest Statement

The authors declare that the research was conducted in the absence of any commercial or financial relationships that could be construed as a potential conflict of interest.
